# Case Report: Surgical management of idiopathic pulmonary aneurysms and review surgical approaches

**DOI:** 10.3389/fcvm.2023.1331982

**Published:** 2023-12-20

**Authors:** Kui Wu, Xuan Fan, Xuanyi Hu, Xuejun Li, Siyuan Yang

**Affiliations:** ^1^ Department of Cardiovascular Surgery, The Affiliated Hospital of Guizhou Medical University, Guiyang, China; ^2^ Department of Surgery, Guizhou Provincial Corps Hospital of Chinese People’s Armed Police Forces, Guiyang, China

**Keywords:** pulmonary aneurysms, idiopathic, procedure, vascular replacement, case report

## Abstract

Idiopathic pulmonary aneurysm is a clinically rare condition characterized by an unknown etiology and episodic occurrence. Despite its rarity, idiopathic pulmonary artery aneurysm poses potential risks to patients. Currently, there is a lack of established clinical guidelines and consensus regarding its management, leading to ongoing controversies in treatment strategies. Particularly, the optimal approach for addressing the main pulmonary artery, its branches, and the pulmonary artery valve remains uncertain. A 57-year-old female patient presented with chest pain and tightness, leading to the diagnosis of idiopathic pulmonary artery aneurysm after excluding other potential causes. Subsequently, she underwent surgical treatment. However, during the surgery, the pulmonary artery wall was found to be extremely weak, prompting us to employ a surgical approach involving the utilization of autologous vessel wrapping with artificial grafts. By summarizing almost all surgical treatment strategies reported in recent years, including the management of pulmonary artery vessels and the pulmonary valve, we have developed a treatment flow chart. This flowchart serves as a valuable guide for the management of future cases presenting similar challenges, offering clinicians valuable insights and evidence-based recommendations.

## 1. Introduction

Pulmonary artery aneurysms (PAAs) are considered to be a rare disease. Deterling and Clagett (1) discovered 8 cases of PAAs in a series of 109,571 consecutive postmortem examinations, resulting in a prevalence rate of 0.0073%. Regarding the definition of the size of pulmonary aneurysms, a population-based survey by Berger et al. (2) reported that the mean diameter of pulmonary arteries (PAs) in a healthy population was 32.0 ± 4.6 mm and suggested that the threshold definition of PAAs should not be less than 45 mm. The etiology of PAAs can be diverse and encompass various factors. These include congenital heart defects, connective tissue abnormalities (e.g., Marfan's syndrome), infectious diseases (e.g., syphilis, tuberculosis, suppurative bacterial infections, and fungal pneumonia), vasculitis (e.g., leukoaraiosis), idiopathic pulmonary arterial hypertension, chronic pulmonary embolisms, neoplasms (e.g., primary lung cancers and lung metastases, medically induced cardiac surgery, as well as the unexplained (idiopathic) cause (3).

Idiopathic PAAs is a rare and mysterious condition characterized by an aneurysm in the pulmonary artery without any known cause. To offer a more precise characterization of idiopathic pulmonary aneurysms, four distinct pathological criteria have been established. (i) dilation of the pulmonary trunk, with or without dilation of other arteries; (ii) the absence of abnormal shunts within or outside the heart; (iii) the absence of chronic cardiorespiratory disease confirmed through clinical or autopsy findings; and (iv) the absence of arterial diseases like syphilis, significant atherosclerosis, or small arteriosclerosis (4). The current diagnostic criteria require the exclusion of these underlying causes to determine the presence of pulmonary artery dilation. Idiopathic PAAs typically manifest as asymptomatic, occasionally present with hemoptysis, and have been reported to result in compression of coronary vessels or the superior vena cava (5–7). For proximal pulmonary aneurysms, conservative treatment is usually used. As aneurysm size increases, surgical resection or graft repair may need to be considered.

Currently, there are no established guidelines for the diagnosis and treatment of PAAs. Although, some literature suggests that conservative treatment has shown favorable clinical outcomes (8, 9). However, large pulmonary aneurysms still pose significant risks, and surgical intervention has been found to provide considerable clinical benefits for patients. In this report, we present a case of surgical treatment for idiopathic PAAs, providing a detailed account of the treatment course and outcomes. Additionally, we review the existing literature on the surgical approach to proximal pulmonary aneurysms.

## 2. Case presentation

**Figure 1:**
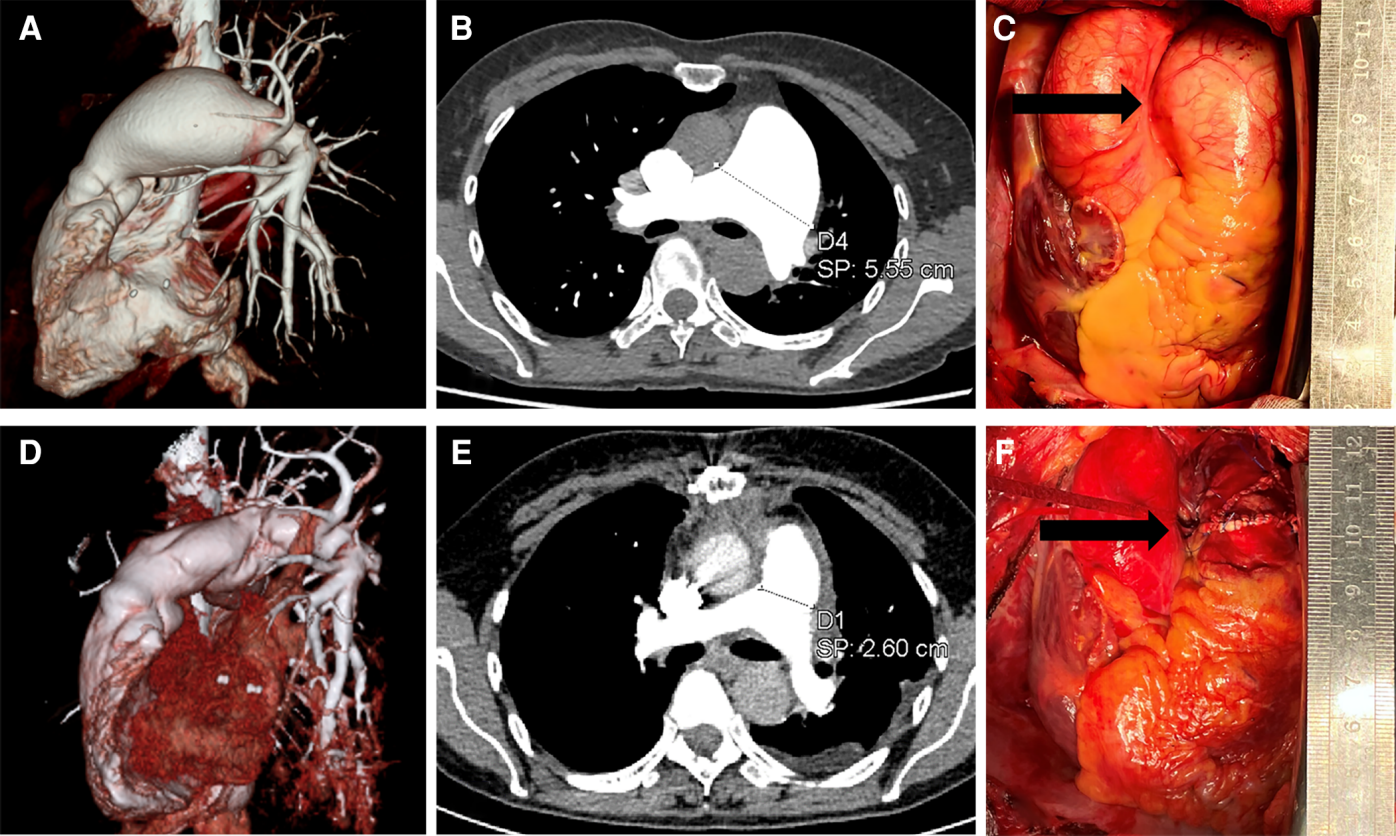
Preoperative and postoperative data. (**A**,**B**) are preoperative pulmonary artery CTA images showing an aneurysmal dilation of the pulmonary artery, with a maximum width of 5.55 cm. (**D**,**E**) are postoperative pulmonary artery CTA images demonstrating restoration of normal diameter in the main pulmonary artery, approximately 2.6 cm. Images (**C**,**F**) are intraoperative images, with (**C**) showing the pulmonary artery aneurysm under direct visualization, and (**F**) displaying the pulmonary artery after vessel replacement.

A 57-year-old woman visited community hospital due to experiencing chest tightness and chest pain for over a year. A chest computed tomography (CT) scan revealed aneurysmal dilation of the pulmonary artery. She had a history of tuberculosis over 20 years ago but stated that she had been successfully treated and cured. During the examination, the patient's blood pressure was measured at 110/70 mmHg, and her pulse rate was recorded at 73 beats per minute. She has a height of 155 cm and a weight of 45 kg. The computed tomography angiography (CTA) scan of the pulmonary arteries revealed significant aneurysmal dilation of the main pulmonary artery and the bifurcation lumen (about 5.6 cm at the wider part), which was considered to be a pulmonary aneurysm; the walls of the main pulmonary artery and its branches appeared smooth and continuous, with no apparent filling defect within the lumen (Figures 1A,B). The electrocardiogram (ECG) displayed a normal sinus rhythm. The echocardiogram reveals no abnormalities in the valves, and there are no abnormal *in vivo* or *in vitro* shunts present. Additionally, there is aneurysmal dilatation of the pulmonary arteries (Supplementary Figure S1). Right heart catheterization revealed a pulmonary artery pressure of 21/11/14 mmHg. Coronary angiography suggests no abnormality. Relevant preoperative routine blood tests are shown in Supplementary Table S1, which were mainly positive for Mycobacterium tuberculosis CD4+ T cells. We recommended surgical treatment of the pulmonary aneurysm. Intraoperatively, it was noted that the main pulmonary artery exhibited significant dilatation from 2 cm above its origin to the bifurcation of the right and left pulmonary arteries, with a maximum diameter of approximately 5.5 cm, and no palpable thrill was detected at the root, and there was a slight dilation observed in the left and right pulmonary arteries (Figure 1C). Cardiopulmonary bypass was initiated, and a sequential blockade of the superior vena cava and inferior vena cava, as well as the right and left pulmonary arteries, was implemented. After the aortic occlusion, cardiac arrest is induced by the infusion of a specialized cardiac arrest solution, effectively ceasing the heart's activity. Intraoperative probes reveal that the pulmonary arteries have thin walls without any signs of dissection or thrombosis. The pulmonary valve annulus is not dilated, and there is no significant regurgitation observed. The aneurysm of the main pulmonary artery was dissected longitudinally and replaced using an artificial blood vessel (24 mm in diameter), which was anastomosed to the proximal and distal ends of the main pulmonary artery, respectively, and the patient's own dilated portion of the pulmonary artery wall was partially excised. The artificial blood vessel was re-sutured and wrapped (Figure 1F). Postoperatively, the patient was returned to the intensive care unit. The patient was transferred to a regular ward on the second day after the surgery and was discharged smoothly one week postoperatively. The diseased vessel was excised and sent for routine pathological examination. Pathological findings showed thinning of the pulmonary artery wall with no other significant abnormalities (Supplementary Figure S2). Postoperative follow-up pulmonary artery CTA showed that the diameter of the main pulmonary artery returned to normal (Figures 1D,E). Anticoagulation with warfarin for 6 months. The patient came for a follow-up visit at the outpatient clinic 2 months later, reporting satisfactory recovery and no specific complaints of discomfort.

## 3. Discussion

In our clinical practice, we have encountered patients with pulmonary aneurysms whose causes could be determined, including two cases of pulmonary aneurysms caused by congenital heart disease (Supplementary Figure S3). However, idiopathic pulmonary aneurysms are considered to be rare. The patient was admitted to the hospital due to chest pain, and subsequent examination eliminated the potential cause of a pulmonary aneurysm. The patient had a previous history of tuberculosis over two decades ago, which had resolved over time. Upon admission, the patient's CD4+ T-cell test for tuberculosis antigen specificity yielded positive results, indicating a prior tuberculosis infection. However, it is important to note that pulmonary aneurysms resulting from tuberculosis infection typically manifest in the distal pulmonary arteries and are not commonly associated with enlargement of the main pulmonary arteries (10). Throughout the surgical procedure, we observed the following details. (i) Preoperative evaluation of the thickness of the pulmonary artery aneurysm vessels is necessary. We observed that the pulmonary arteries exhibited a marked thinness, measuring approximately 1 mm. Despite the absence of elevated pulmonary artery pressure, we chose to use the patient's blood vessel to wrap the artificial blood vessel as a precautionary measure for enhanced safety. Nonetheless, during the distal anastomosis, we noted that the vessel walls remained susceptible to tearing due to their thin and fragile condition. Consequently, we fortified the vessel walls by applying an external vascular patch. (ii) To mitigate the risk of bleeding, we advised complete exposure of the bilateral pulmonary arteries before the surgery and temporary occlusion using polyester tapes. This approach facilitated a more optimal surgical field of vision. (iii) Regarding whether to stop the heartbeat, we opted for a “stopped” approach during the surgery to ensure a clearer view for the patient, considering the blood reflux from the coronary sinus. (iv) Regarding the selection of anticoagulation therapy, our standard practice following ascending aorta artificial graft surgery involves the administration of aspirin for 3–6 months. However, considering the slower blood flow velocity in the pulmonary artery compared to the aortic side and the absence of comprehensive anticoagulation protocols in existing literature, we have opted for a warfarin anticoagulation regimen for 3–6 months to prioritize patient safety.

We conducted a comprehensive analysis of recent cases involving idiopathic pulmonary aneurysms and examined the surgical techniques employed in these cases (Supplementary Table S2). Previous studies primarily focused on discussing the indications for surgery, with limited discussion on the available surgical treatment options and choices. By integrating patient cases and conducting a thorough literature review, we have compiled a summary of the various surgical treatment options and choices available for this condition.

**Figure 2:**
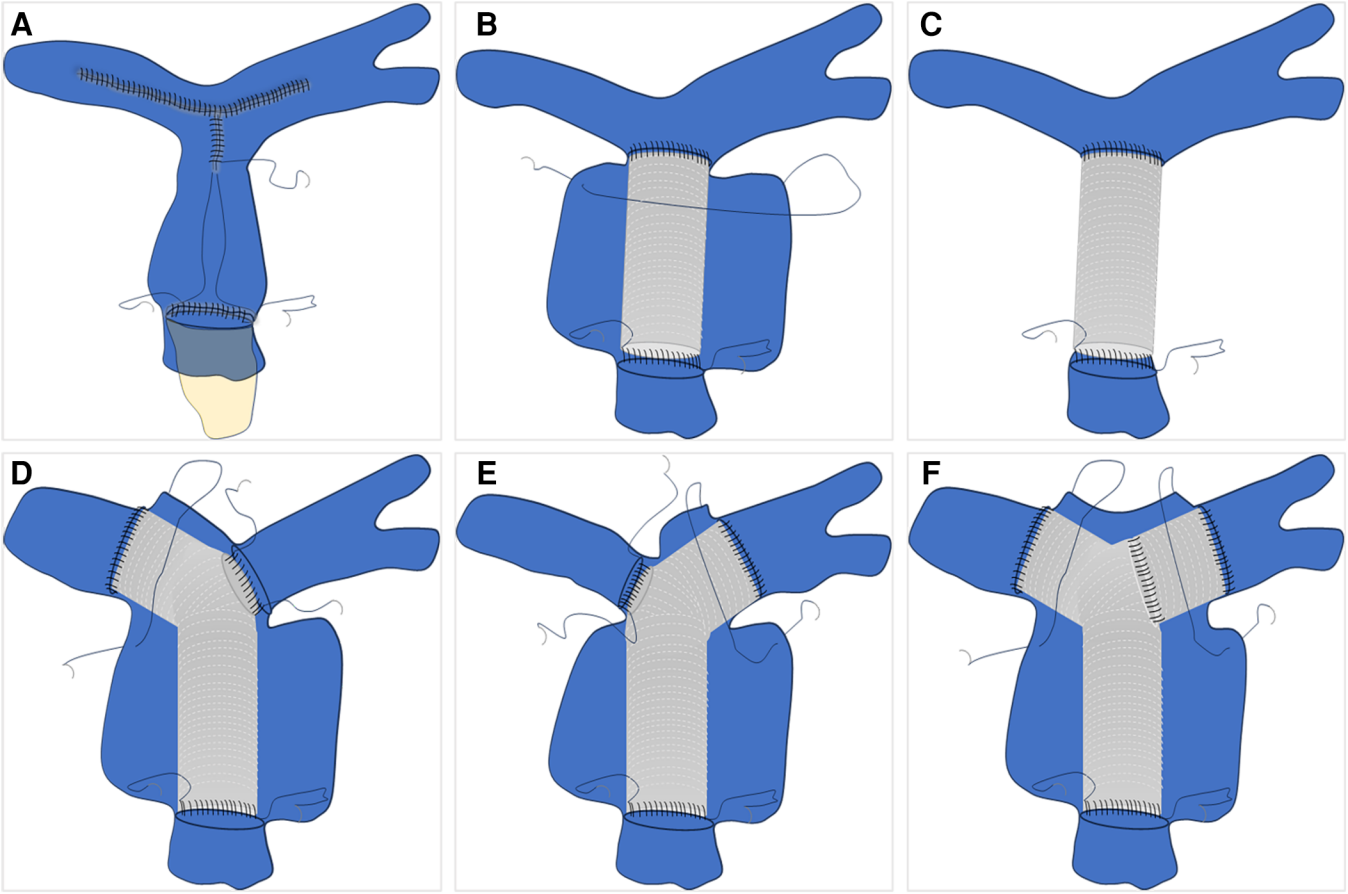
Pulmonary artery vascular management schematic. (**A**) Direct excision and suturing of the dilated pulmonary artery. (**B**) Replacement of the ascending pulmonary artery with an artificial blood vessel and wrapping. (**C**) Replacement of the ascending pulmonary artery with an artificial blood vessel without autologous vessel wrapping. (**D**–**F**) represent schematic illustrations of involvement of the left pulmonary artery, right pulmonary artery, or bilateral pulmonary artery branches, respectively, with subsequent replacement.

**Figure 3:**
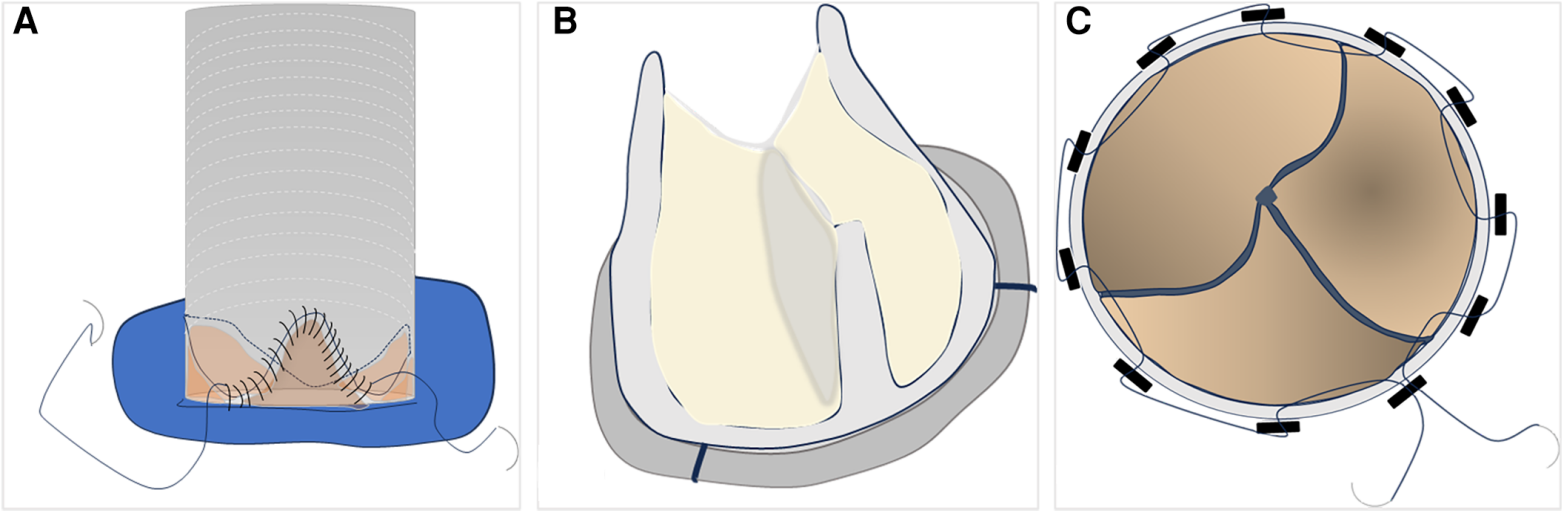
Schematic illustrations of pulmonary artery management. (**A**) Similar to the David procedure; (**B**) bioprosthetic valve replacement; (**C**) De Vega-like procedure.

The initial step is to assess whether the patient meets the necessary indications for surgery. For indications of surgery for pulmonary aneurysms, Kreibich et al. (3) suggest the following factors be considered: (Ⅰ) absolute aneurysm diameter ≥5.5 cm; (Ⅱ) increase in aneurysm diameter of ≥0.5 cm within 6 months; (Ⅲ) compression of adjacent structures; (Ⅳ) formation of a thrombus within the aneurysm; (Ⅴ) development of clinical symptoms; (Ⅵ) presence of valvulopathy or shunts; (Ⅶ) confirmed diagnosis of pulmonary arterial hypertension; and (Ⅷ) rupture of aneurysm or sandwich formation. The second step involves managing the pulmonary artery vasculature. Firstly, the thickness of the vessel wall needs to be assessed. For patients with appropriate or normal vessel wall thickness, direct removal of the pulmonary aneurysm can be performed. However, for patients with very thin pulmonary arteries, it is recommended to use artificial vascular grafts, along with wrapping using the patient's blood vessels (11–13). Subsequently, the involvement of both the right and left pulmonary arteries must be taken into consideration. In cases where the pulmonary arteries are cumulatively involved or bilaterally involved, simultaneous vascular replacement on one or both sides may be necessary (14–17) (Figure 2). The last step involves considering the management of the pulmonary valve. The functionality of the pulmonary valve needs to be evaluated to determine if it is affected. If the valve function is normal and only the annulus is enlarged, a procedure similar to David's (16) or Devega's (15) can be performed, preserving as much function of the pulmonary valve as possible; if the valve function is abnormal, a bioprosthetic pulmonary valve is also a good option (12, 18) (Figure 3).

**Figure 4:**
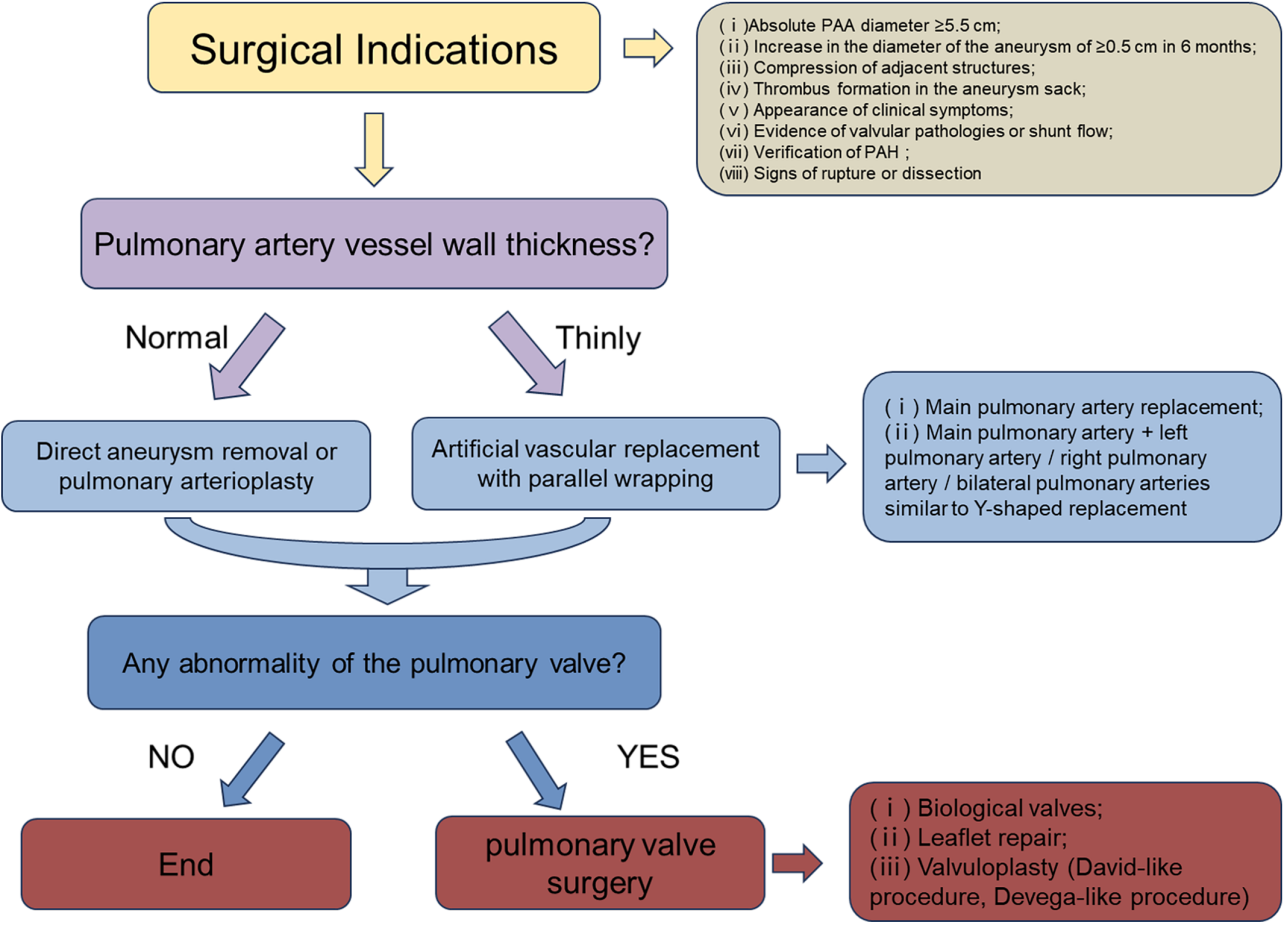
Flowchart of surgical treatment modalities for idiopathic pulmonary aneurysms.

We have devised a treatment flowchart (Figure 4) exclusively applicable to patients diagnosed with idiopathic pulmonary aneurysm, excluding those with secondary pulmonary artery dilatation. This flowchart is intended to serve as a comprehensive guide for subsequent clinical management and decision-making processes.

## 4. Conclusion

Surgical treatment of Idiopathic PAAs is comparatively safe and clinically beneficial for postoperative patients. However, it requires attention to a series of details and selection of appropriate surgical techniques. By creating a flowchart, we can provide valuable clinical thinking and procedures for subsequent similar patients, thereby improving the accuracy and effectiveness of treatment and providing better medical care.

## 5. Patient perspective

“I had no prior knowledge about idiopathic pulmonary artery, the type of disease I was diagnosed with. However, I consider myself fortunate to have received timely surgical intervention, which ensured my safety. I am deeply grateful to the medical staff for their exceptional treatment. I sincerely hope that my experience can serve as a valuable reference for similar cases”. We appreciate the patient's encouragement and support in reporting the article!

## Data Availability

The original contributions presented in the study are included in the article/**Supplementary Material**, further inquiries can be directed to the corresponding authors.
