# Supplementary material for: Case Report: Surgical management of idiopathic pulmonary aneurysms and review surgical approaches
Source: Front Cardiovasc Med. 2023 Dec 20;10:1331982. doi: 10.3389/fcvm.2023.1331982 (PMC10761405; doi:10.3389/fcvm.2023.1331982)
Supplement: Supplementary file 1 [file Datasheet1.docx]

Supplementary Material

# Supplementary Figures and Tables

## Supplementary Figures


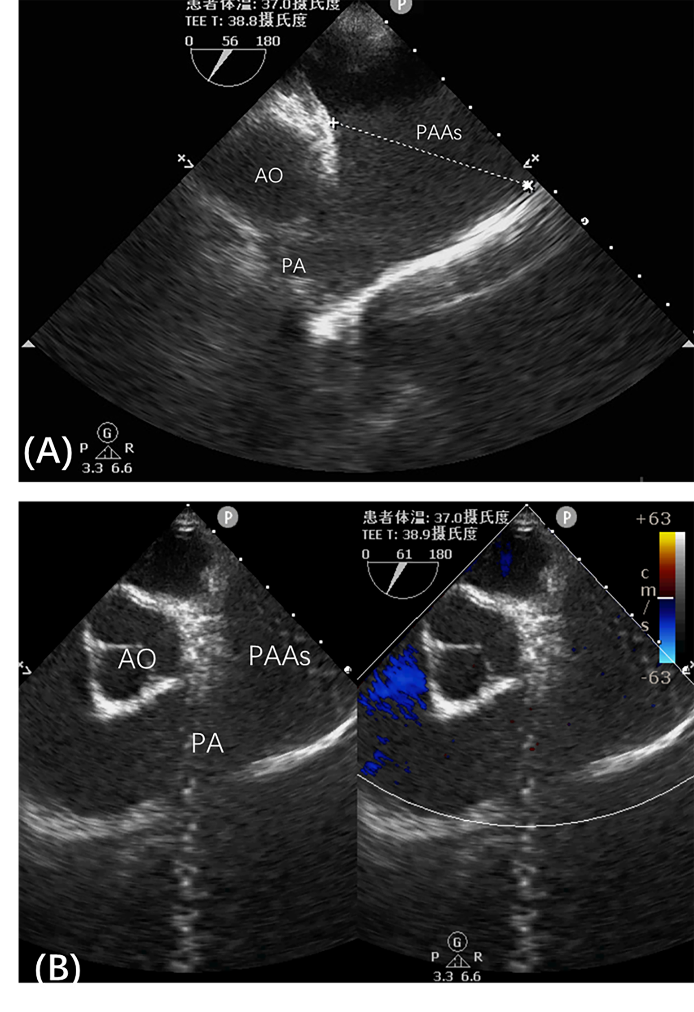


**Supplementary Figure S1.** Echocardiogram. (A) Ultrasound diagram of pulmonary aneurysm. (B) Pulsed-wave Doppler ultrasound image.AO: Aorta; PA: Pulmonary Artery; PAAs: Pulmonary artery aneurysms.


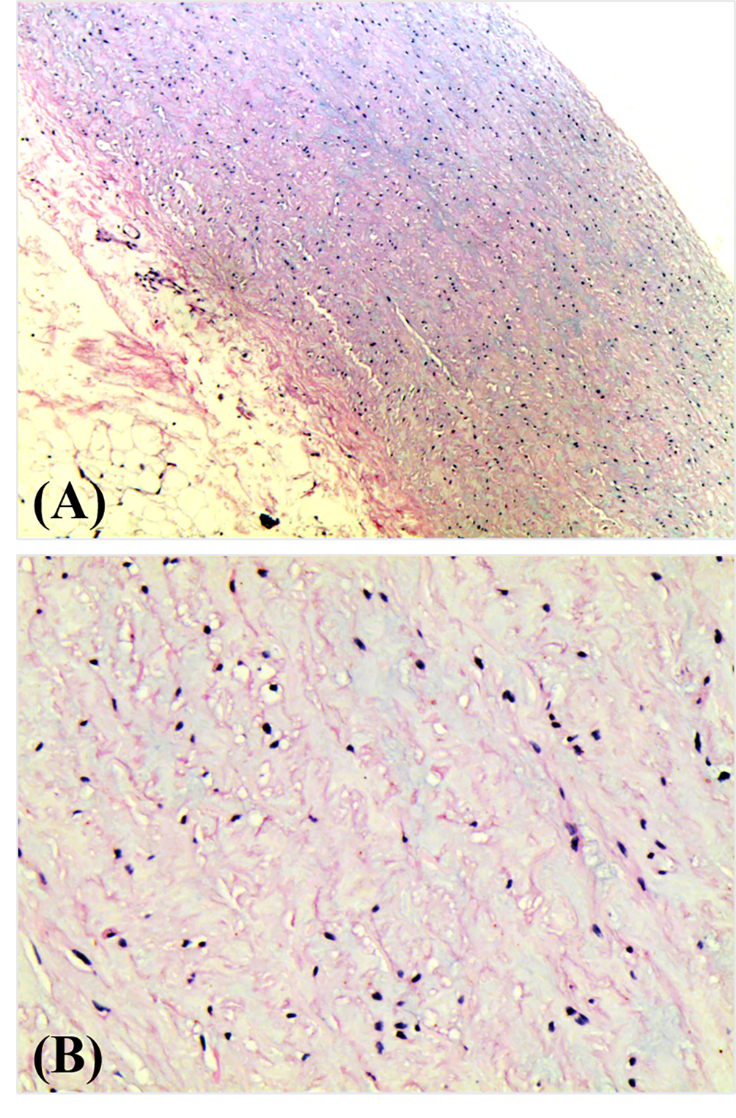


**Supplementary Figure S2.** Pathological sections of idiopathic pulmonary aneurysms. (A) Cross-section of blood vessels showing significant thinning of the vessel wall. (B) No apparent abnormal pathological changes observed.


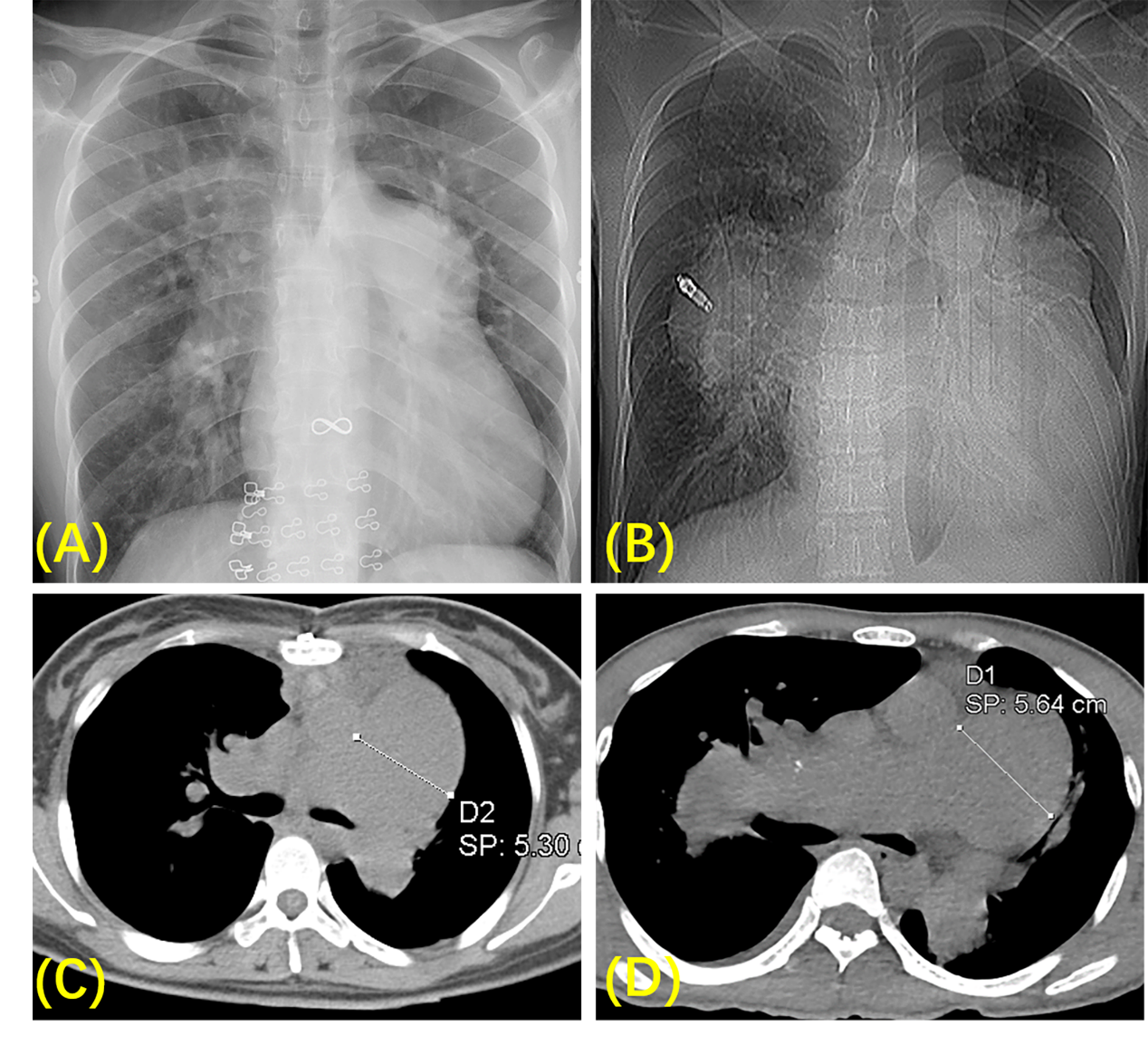


**Supplementary Figure S3.** Pulmonary artery aneurysm-like alterations associated with congenital heart disease. (A) and (C) demonstrate the presence of pulmonary artery aneurysm-like changes in a 35-year-old patient diagnosed with atrial septal defect and severe pulmonary arterial hypertension, with a maximum width measuring 5.30 cm. (B) and (D) illustrate the occurrence of pulmonary artery aneurysm-like changes in a 30-year-old patient diagnosed with ventricular septal defect, pulmonary arterial hypertension, and Eisenmenger syndrome, with a maximum width measuring 5.64 cm. Informed consent has been obtained from the patients for publication.

## Supplementary Tables

**Table S1. Lab findings on presentation**

| **Labs** | **Results** | **Reference range** |
| --- | --- | --- |
| C-reactive protein (CRP) | 4.08mg/L | ＜5 |
| Erythrocyte Sedimentation Rate（ESR） | 42 mm/h | 0-38 |
| anti-Cyclic Citrullinated Peptide (CCP) | 6.91 AU/mL | 0-17 |
| IgG | 10.8 g/L | 8.6-17.4 |
| IgA | 2.15 g/L | 1.00-4.20 |
| IgM | 1.11 g/L | 0.50-2.80 |
| Rheumatoid Factor(RF) | ＜9.88IU/mL | 0-15.90 |
| Antisense Oligonucleotides (ASO) | 63.8 IU/mL | 0.00-214.0 |
| Anti-Neutrophil Cytoplasmic Antibody (ANCA) | Negative | Negative |
| (1 → 3)-β-D-glucan | ＜37.5 pg/mL | 0-70(negative) |
| Antinuclear Antibody (ANA) | Negative | Negative |
| Anticardiolipin Antibodies（ACLA） | Negative | Negative |
| Mycobacterium tuberculosis-interferon release assay（TB1-N、TB2-N） | ＞10 IU/mL | ＜0.35 |
| QuantiFERON-TB Gold | Positive | Negative |
| Syphilis Spirochete Antibody | Negative | Negative |

| **Table S2. Summary of case reports of** **pulmonary artery aneurysm** | | | | | | |
| --- | --- | --- | --- | --- | --- | --- |
| Author, Years, Ref. | Age(y)/ gender | MPA (mm) | Branches involvement(mm) | Surgical Procedures | PH (mmHg) | Outcome |
| Xie, 2023, (1) | 51/M | 52 | NO | Aneurysmectomy (MPA); Synthetic Graft (MPA); | No data | CR |
| Flaifel, 2023, (2) | 68/F | 61 | NO | Aneurysmectomy (MPA); Synthetic Graft (MPA); Inclusion Technique | 60 | CR |
| Stevens, 2023, (3) | 76/F | 90 | LPA:30; RPA:30 | Pulmonary Valve Surgery (Biological valves); Aneurysmectomy (MPA); Synthetic Graft (MPA); Inclusion Technique (Dacron tube graft) | No data | CR |
| Badders, 2023, (4) | 61/F | 63 | No | Synthetic Graft (MPA); Inclusion Technique | 27 | CR |
| Salehi, 2022, (5) | 36/M | 55 | LPA:69 | Surgery (No description) | 35 | CR |
| Kultursay, 2022, (6) | 40/F | 106 | No | Surgery (No description) | 50 | CR |
| Qian, 2020, (7) | 59/F | 68 | LPA:26; RPA:54 | Pulmonary Valve Surgery (Biological valves); Aneurysmectomy (MPA+RPA); Synthetic Graft (MPA, RPA); | 37 | CR |
| Hou, 2020, (8) | 47/M | 153 | No | Aneurysmectomy (MPA); Synthetic Graft (MPA); | No data | CR |
| Hong, 2020, (9) | 64/M | 97.3 | LPA:43; RPA:53.7 | Pulmonary Valve Surgery (Devega-like); Aneurysmectomy (MPA, LPA, RPA); | 20 | CR |
| Circi, 2020, (10) | 39/F | 80 | LPA:30; RPA:50 | Pulmonary Valve Surgery (David-like); Aneurysmectomy (MPA, LPA, RPA); | 58 | CR |
| Worku, 2019, (11) | 45/F | 71 | No data | Pulmonary Valve Surgery (Biological valves); Aneurysmectomy (MPA, RPA); Synthetic Graft (MPA, RPA); | 15 | CR |
| Worku, 2019, (11) | 56/F | 61 | No data | Pulmonary Valve Surgery (repair); Aneurysmectomy (MPA); Synthetic Graft (MPA); | 32 | CR |
| Worku, 2019, (11) | 51/F | 68 | No data | Pulmonary Valve Surgery (Biological valves); Aneurysmectomy (MPA); | 85.2 | CR |
| Worku, 2019, (11) | 57/F | 70-80 | No data | Pulmonary Valve Surgery (Biological valves); Aneurysmectomy (RPA); Synthetic Graft (RPA); | 30 | CR |
| Balcı, 2019, (12) | 55/M | 58 | No data | Aneurysmectomy (MPA); Synthetic Graft (MPA); | 20 | CR |
| Kanaoka, 2017, (13) | 68/F | 106 | No data | Pulmonary Valve Surgery (Biological valves); Aneurysmectomy (MPA); | 35 | CR |
| Hou, 2016, (14) | 45/F | 80 | No | Pulmonary Valve Surgery (Biological valves); Aneurysmectomy (MPA); | No data | CR |
| Haj-Yahia, 2018, (15) | 47/M | 45 | LPA:60; RPA:23 | Aneurysmectomy (MPA, LPA, RPA); Synthetic Graft (MPA, LPA, RPA); | No data | CR |
| Seguchi, 2011, (16) | 45/M | 70 | No data | Pulmonary Valve Surgery (Repair); Aneurysmectomy (MPA); Synthetic Graft (MPA); | No data | CR |
| Muthialu, 2010, (17) | 25/F | 60 | No data | Pulmonary Valve Surgery (Repair); Aneurysmectomy (MPA); | No data | CR |
| Salhab, 2007, (18) | 71/M | 70 | LPA involvement | Aneurysmectomy (MPA, LPA); Synthetic Graft (MPA); | 10 | CR |
| Holzinger, 2011, (19) | 60/M | 95 | LPA:47; RPA:35 | Aneurysmectomy (MPA, LPA, RPA); | 11 | CR |

F: Female; M: Male; MPA: Main pulmonary artery; LPA: Left pulmonary artery; RPA: Right pulmonary artery; PH: Pulmonary hypertension CR: Complete remission

References

1. Xie Z, Deng M, Yang Q: Giant idiopathic pulmonary artery aneurysm. *Asian J Surg* 2023, 46(8):3135-3136. https://doi.org/10.1016/j.asjsur.2023.02.083

2. Flaifel M, Suresh Daniel R, Nakanishi H, Than CA, Shiakos G, Tzanavaros I: A Novel Approach for the Treatment of Pulmonary Artery Aneurysm Repair Using Inclusion Technique: A Case Report. *Cureus* 2023, 15(3):e36456. https://doi.org/10.7759/cureus.36456.

3. Stevens M, Swan KW, Bommareddi S, Ali SO: Two giants: Giant cell arteritis causing a giant pulmonary artery aneurysm. *JTCVS Tech* 2023, 20:79-82. https://doi.org/10.1016/j.xjtc.2023.04.016.

4. Badders J, Roughneen P, Mohan N, Roughneen E: Pulmonary Artery Aneurysm: A Rarity and Surgical Enigma. *Cureus.* 2023. https://doi.org/10.7759/cureus.38157.

5. Salehi AM, Khansari N: Idiopathic Pulmonary Artery Aneurysm: A Case Report. *Curr Cardiol Rev* 2022, 18(5):50-52. https://doi.org/10.2174/1573403x18666220428122804.

6. Kultursay B, Keskin B, Karagoz A, Akbal OY, Kaymaz C: Giant pulmonary artery aneurysm caused by sibutramine-associated pulmonary arterial hypertension: First case in the literature. *Anatol J Cardiol* 2021, 25(7):512-514. https://doi.org/10.5152/AnatolJCardiol.2021.64166.

7. Qian Q, Subbian SK, Kofidis T: Total pulmonary artery replacement with an Avalus-Gelweave conduit in a patient with giant pulmonary artery aneurysm with pulmonary regurgitation. *J Card Surg* 2020, 35(5):1122-1124. https://doi.org/10.1111/jocs.14517.

8. Hou J, Fang Y: Giant pulmonary aneurysm. *Anatol J Cardiol* 2020, 23(5):E14. https://doi.org/10.14744/AnatolJCardiol.2020.57598.

9. Hong Son PD, Tu VN, Uoc NH, Vo HL: Successful Aneurysmorrhaphy for a Giant Idiopathic Pulmonary Artery Aneurysm. *Innovations (Phila)* 2020, 15(3):275-278. https://doi.org/10.1177/1556984520911667.

10. Circi R, Boysan E, Behlul Altunkeser B, Aygul N, Cagli K, Cagli K, Sener E: David's procedure for pulmonary artery aneurysm. *J Card Surg* 2020, 35(4):942-945. https://doi.org/10.1111/jocs.14480.

11. Worku BM, de Angelis P, Wingo ME, Leonard JR, Khan FM, Hameed I, Ruan Y, Gaudino MFL, Girardi LN: Pulmonary artery aneurysms: Preoperative, intraoperative, and postoperative findings. *J Card Surg* 2019, 34(7):570-576. https://doi.org/10.1111/jocs.14070.

12. Balcı E, Demir A, Özgök A, Karadeniz Ü: Anaesthesia Management in Idiopathic Pulmonary Artery Aneurysm Surgery. *Turk J Anaesthesiol Reanim* 2019, 47(6):503-506. https://doi.org/10.5152/tjar.2019.21447.

13. Kanaoka K, Horii M, Nagato H, Kaneda K: A giant pulmonary artery aneurysm. *Eur Heart J Cardiovasc Imaging* 2018, 19(2):236. https://doi.org/10.1093/ehjci/jex233.

14. Hou R, Ma GT, Liu XR, Zhang CJ, Liu JZ, Cao LH, Li XF, Miao Q: Surgical treatment of pulmonary artery aneurysm: an institutional experience and literature review. *Interact Cardiovasc Thorac Surg* 2016, 23(3):438-442. https://doi.org/10.1093/icvts/ivw157.

15. Haj-Yahia S, Sbaih M, Bali K, Darwazah A, Othman W, Zaghari M, Angelini G, Caputo M, Barqawi AK: Case report and management approach in idiopathic pulmonary arteries aneurysm. *J Cardiothorac Surg* 2018, 13(1):110. https://doi.org/10.1186/s13019-018-0791-9.

16. Seguchi M, Wada H, Sakakura K, Kubo N, Ikeda N, Sugawara Y, Yamaguchi A, Ako J, Momomura S: Idiopathic pulmonary artery aneurysm. *Circulation* 2011, 124(14):e369-370. https://doi.org/10.1161/circulationaha.111.029033.

17. Muthialu N, Raju V, Muthubaskaran V, Chandrasekar P, Muralidharan S, Kuppanna PJ: Idiopathic pulmonary artery aneurysm with pulmonary regurgitation. *Ann Thorac Surg* 2010, 90(6):2049-2051. https://doi.org/10.1016/j.athoracsur.2010.05.071.

18. Salhab K, McLarty A: Idiopathic pulmonary artery aneurysm. *Thorac Cardiovasc Surg* 2007, 55(5):329-331. https://doi.org/10.1055/s-2006-955909.

19. Holzinger C, Podesser BK, Lomoschitz F, Kassal H: Idiopathic pulmonary artery aneurysm. *J Card Surg* 2011, 26(2):154-156. https://doi.org/10.1111/j.1540-8191.2010.01166.x.
