# Supplementary figures and images for: Case Report: Surgical management of idiopathic pulmonary aneurysms and review surgical approaches
Source: Front Cardiovasc Med. 2023 Dec 20;10:1331982. doi: 10.3389/fcvm.2023.1331982 (PMC10761405; doi:10.3389/fcvm.2023.1331982)

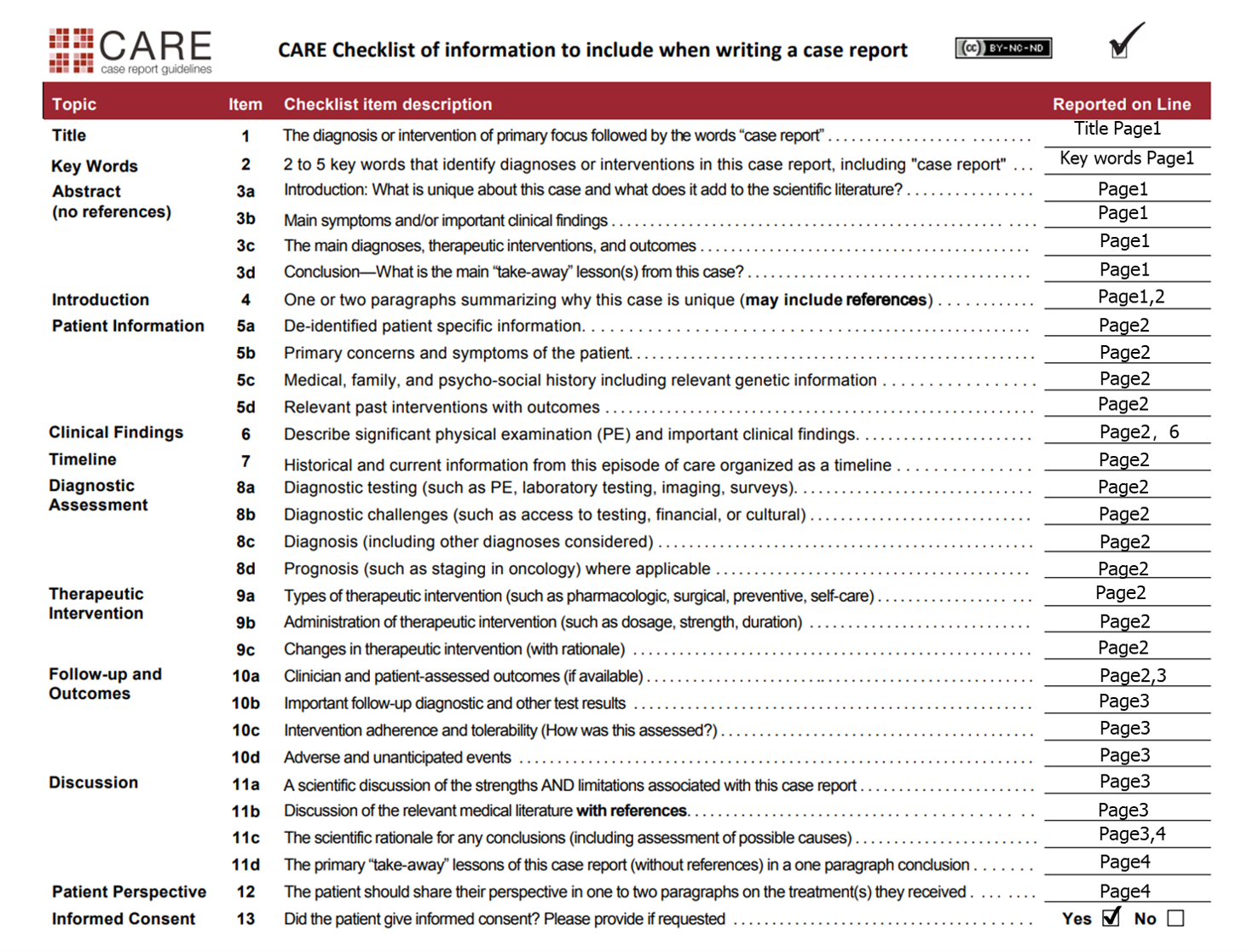

Supplement: Supplementary file 2 [file Image1.tif]
